# Gynecological and Obstetrical Society of Maryland

**Published:** 1892-03

**Authors:** William S. Gardner

**Affiliations:** Secretary; 712 N. Howard St.


### GYNECOLOGICAL AND OBSTETRICAL SOCIETY OF MARYLAND.

---

### JANUARY MEETING.

The President, W. E. Moseley, in the chair.

Dr. T. A. Ashby exhibited a specimen of a dermoid cyst which he had recently removed from a single woman 25 years of age. The cyst grew from the left ovary, and had been diagnosed as ovarian cyst. It measured 4 1-2 by 5 1-2 inches in diameter. Its removal was accomplished without trouble, and the patient made prompt recovery.

The interest in the case centered in the character of the cyst, and its contents. It was lined internally with dermal tissue, and at one point the layer covered a small piece of bone. At this point a long twist of hair grew from the skin and measured thirty inches in length. The cyst contained a large collection of sebacious and oily matter, and large strands of hair disconnected from the tumor and matted together in the cyst.

The specimen is an uncommon variety of dermoid cyst. A similar case has been reported by Dr. Munde, of New York. Dr. Ashby referred brifly to dermoid tumors.

They are no doubt due to an irregular derangement of the epiblastic layer of the tissues of the fœtus in embryonic life. The cysts are almost uniformly found in organs and tissues which owe their origin to the layer of the epiblast, and this circumstance goes to explain the peculiar features which they present. The ovary is a very common location for them, and this fact would tend to prove that the organs of generation originate in the epiblast.

Dr. Wilmer Brinton read a paper on "From Pregnancy, Complicated by Placenta Previa Centralis."

I was summoned at 3:30 o'clock on the morning of September 1st, 1891, to see Mrs. B. T. M. Her second confinement from her reckoning, would take place October 15th. I did not see her until sent for, as stated, on the morning of September 1st. Upon arriving at her home, I found my patient in bed with the history of being awakened some time before I was sent for, by having a few sharp pains, which were followed by a profuse hemorrhage. Upon examining Mrs. M., I found the vagina filled with large clots of blood, the os slightly dilated and very soft, and a placenta presenting. At this time, her pains having ceased, I cleaned out the clots from the vagina, and found the bleeding had stopped. I determined to return home, which I did, leaving orders for my patient to remain quiet, and to send for me at once if the pains or bleeding returned. I was informed before leaving the house that a week previous the patient had had a severe hemorrhage, and knowing I was out of the city, Dr. W. J. Jones, who lives in the immediate vicinity, was sent for. He saw her twice, and, under the treatment and advice he gave her, the bleeding ceased. At 6:30, or about three hours from the time I left the house, I was again sent for. I immediately responded and was soon joined, at my request, by my colleague, Dr. Crouch, and by Dr. J. H. Robinson.

A vaginal examination made at this time found the vagina filled with blood, which was continuing to flow. The os was more dilated and dilatable than it was at my previous examination, and a more complete examination found the placenta presenting, which was of the most complete central variety which I had ever seen. In running my finger around, I found the placenta was completely attached to the mucous membrane of the lower segment of the uterus, with the exception of a small space on the left side, from which the bleeding came, and in which a tear had taken place during the recent contrac tions which had severed a small portion of the placenta from the attachments. My opinion being verified by the gentlemen present, and as the hemorrhage and pains were continuing, we determined to deliver at once. Chloroform was administered, and introducing my hand, I found the cervix not well dilated, and had some trouble in introducing my hand. I tore rapidly through the placenta at the left side, and found a child presenting vertex. I ruptured the bag of water, and delivered it living by podalic version. In my efforts to do this, I was made conscious for the first time that the uterus contained a second child; so, tying the cord of the first child, and handing it to Dr. Crouch, who was ably assisting me, I introduced my hand for the second time and found the second child presenting shoulder, "*dorso anterior position*," the head being to the mother's left. I turned and immediately delivered a second living child, after which I introduced my hand into the uterus and removed the placenta, which presented a very ragged appearance from my efforts made in passing it at its attachment to the uterus on the left side. After removing the placenta, the vagina and uterus were thoroughly washed out with warm water, during which I discovered the cervix was lacerated on both sides, due, no doubt, to my efforts to deliver the children through an imperfectly delated cervix. Although a large amount of blood was lost during the operative procedures, the woman rallied well from the chloroform, the uterus contracted well, and our patient, within a few hours, presented no special traces of the severe ordeal she had passed through. The children, both males, presented the appearance of having advanced to the seven and a half months' utero gestation, and for two or three hours after birth did well, but later on in the day their extremities became cold, lips blue, heart weak, and they died some seven hours after their birth. The mother did fairly well for about one week, although the pulse and temperature were somewhat above normal—the pulse averaging between 90 and 100, the temperature being about 100. She sat up on the eleventh day, and on the following day I was sent for, and found her with a high temperature and rapid pulse, with some indications of "phlegmasia alba dolens," and for three weeks she was under my constant care, with evidence of well marked septic complications, and as soon as the tendency for the phlebitis disappeared in one leg, it appeared in the other.

I am satisfied the late septic complications occurred from the lacerated cervix, which healed up kindly on the right side, but not so on the left, which healed slowly by granulations.

Upon my recall to the case on the 11th day, I took charge of the vaginal injections myself. Previous to this time, I had entrusted this to the nurse, much to my regret, for upon my first examination, I was satisfied they had not been thoroughly given ; so every day for several days, I introduced a speculum and with an ordinary piston syringe, I washed out the uterus, the cervix and the vagina, with bi-chloride or carbolic acid sol. and dusted the seat of laceration with either boracic acid or iodoform. Internally was given quinine, phenacetine, large doses of iron, and good food.

The leg was bandaged from time to time with an ordinary roller bandage. Greatly to my relief, my patient finally recovered, and seven weeks after her confinement, returned to her home in Washington.

My object in reporting this case is to impress on the minds of physicians the importance of not temporizing when they have to do with a case of placenta previa.

There is no safety for the mother as long as she remains undelivered. I am satisfied no one can lay down dogmatic rules in every individual case, but my own personal experience has taught me that in performing podalic version, and delivering either rapidly or slowly as the case may indicate, you are working for the best interest of the mother and child, in the majority of cases.

Dr. Wm. P. Chunn.—I have seen only two cases of placenta previa. One I saw with Dr. Neal. The patient had been tamponed with cotton. He took out the cotton, inserted his left hand, and delivered the child by podalic version. Both mother and child did well. I had one patient of my own. It was a marginal implantation, and I thought I could use the forceps better than turn, and I did so. I had some difficulty in getting the forceps on, and failed at first, but the attending physician forced the head firmly down, by external pressure the forceps were put on and the child delivered. I think I might have done better by podalic version.

Dr. Brinton.—There is no absolute law for the treatment of placenta previa. In my first case the patient was lost by delay. In another case, the woman had bled considerably, but about the time I was called the head came down and the bleeding stopped.

Forceps were put on and the child delivered. I am now satisfied that the first patient could have been saved by prompt action.

In the ten cases of placenta previa which I have seen in practice, only two of the children have been saved. The mothers have all recovered with the one exception as specified above

Dr. T. A. Ashby.—I think Dr. Brinton did the proper thing in this case. My experience with these cases has been limited, having seen but two. In one, the child was born dead. The mother recovered. The placenta was attached over the entire cervix, and had to be torn away before the child could be delivered.

In the second case, I removed a dead fœtus of five or six months with placenta previa. She had been bleeding for some weeks. She recovered and subsequently gave bir h to a living child. More recently I delivered her of another dead fœtus.

With reference to the therapeutic trouble which the doctor's patient had suffered from, I am satisfied that lacerated cervix is a prolific cause of pelvic troubles, and I frequently find laceration of the cervix and involvement of the tubers associated.

The treatment that the doctor suggested, of going into the uterus and washing it out thoroughly, is very good. My own method is somewhat different; I put in a speculum, fill up the vagina with a bichloride solution, and then with some cotton, on an application remove all the debris from the cavity of the uterus. I have treated eight cases in this way in the last year, and in each case got a good result.

I have seen but one case of pure septicæmia, that came on four weeks after confinement. There were no local lesions, and there was nothing in the uterus to be removed. The symptoms came on the twenty-first day after confinement, and she died in about a week.

WILLIAM S. GARDNER, M. D., Secretary.

712 N. Howard St.

